# 1439. Identifying Barriers and Improving Measures for Hand Hygiene Practices Among Healthcare Workers in Korea: A Cross-Sectional Survey

**DOI:** 10.1093/ofid/ofad500.1276

**Published:** 2023-11-27

**Authors:** Se Yoon Park, Jaewoong Kim, Shi Nae Yu, Yeon Su Jeong, Jin Hwa Kim, Tark Kim, Min Hyok Jeon, Eun Ju Choo, Eunjung Lee, Tae Hyong Kim

**Affiliations:** Hanyang University College of Medicine, Yongin, Kyonggi-do, Republic of Korea; Yonsei University College of Medicine, Seoul, Seoul-t'ukpyolsi, Republic of Korea; Soonchunhyang University College of Medicine, Cheonan, Ch'ungch'ong-namdo, Republic of Korea; Soonchunhyang University Seoul Hospital, Seoul, Seoul-t'ukpyolsi, Republic of Korea; Soonchunhyang University Seoul Hospital, Seoul, Seoul-t'ukpyolsi, Republic of Korea; Department of Internal Medicine, Soonchunhyang University Bucheon Hospital, Bucheon, Kyonggi-do, Republic of Korea; Soonchunhyang University College of Medicine, Cheonan, Ch'ungch'ong-namdo, Republic of Korea; Soonchunhyang University Bucheon Hospital, Bucheon, Kyonggi-do, Republic of Korea; Soonchunhyang University Seoul Hospital, Seoul, Korea, Seoul, Seoul-t'ukpyolsi, Republic of Korea; Division of Infectious Diseases, Department of Internal Medicine, Soonchunhyang University Seoul Hospital, Seoul, Seoul-t'ukpyolsi, Republic of Korea

## Abstract

**Background:**

Hand hygiene (HH) is a fundamental component of infection prevention and control in healthcare settings. This study aimed to identify barriers to HH according to occupational group to increase the rate of HH compliance among healthcare workers (HCWs).

**Methods:**

This cross-sectional survey was conducted in July 2018 at four university affiliated hospitals. The survey comprised seven parts with 49 items, including self-reported HH compliance, knowledge, attitudes, behaviours, barriers to HH, and improvement strategies.

**Results:**

A total of 1,046 HCWs participated in the survey. The self-reported HH compliance rate was highest in the nurses group, followed by other HCWs and physicians (Figure 1). The scores regarding knowledge, attitudes, and behaviours about HH were highest in the nurses group. The nurses group also had higher scores in terms of internal and emotional motivation. The most important intervention was “hand sanitizer placed where necessary” followed by “regular HH education” and “reward and publicize excellent HH employees/departments”. The distribution of the nurses, physicians, and other HCWs was similar, and a downward bias can be seen for the physicians (Figure 2). In emergency situations, physicians and nurses found HH the most challenging, while other HCWs considered skin problems caused by HH products the most significant barrier. Among 12 improvement measures, around 20% of the respondents ranked "diversify types of hand sanitizers", "install soap and paper towels in each hospital room", and "change perception through various HH campaigns" as the top three priorities. The physician group deemed the timely reminder of HH compliance as the second most critical improvement measure (Figure 3).

Hand hygiene and optimal hand hygiene compliance rate.

**Figure:**
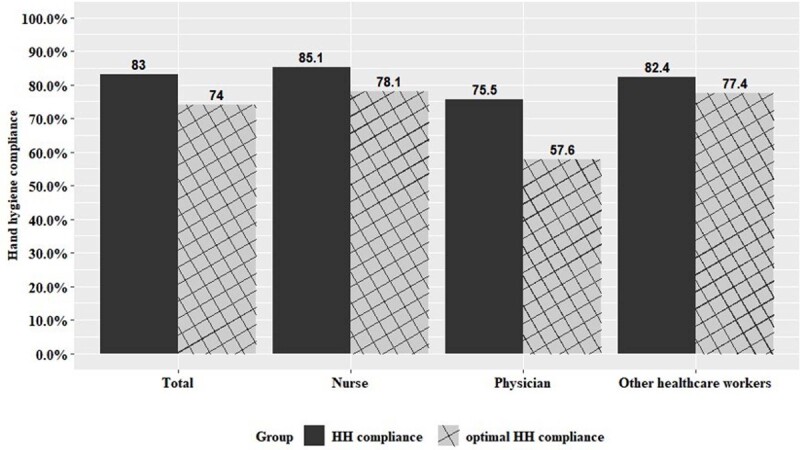

Relationship between importance and achievement scores for hand hygiene improvement measures.

**Figure:**
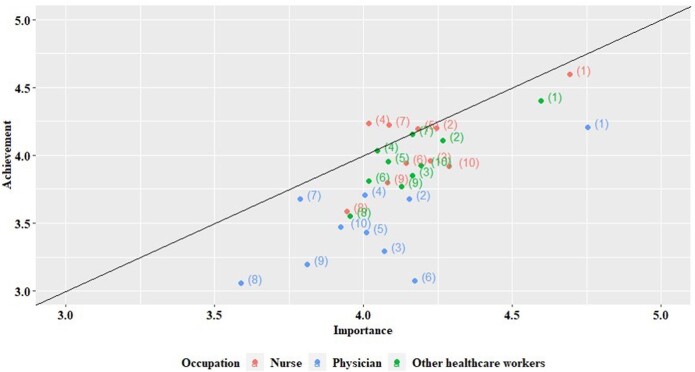

The graph shows the importance and achievement scores for each measure on a scale of 1-5, with 1 being low and 5 being high. The measures include hand sanitizer placed where necessary (1), regular hand hygiene education (2), practical training according to the situation (3), frequent monitoring (4), department-wide feedback (5), personal feedback (6), hand hygiene information poster (7), audiovisual alarming/guidance (8), management’s interest and encouragement (9), and reward and publicize excellent hand hygiene employees/departments (10).

Improvement measures for barriers to performing hand hygiene based on first choice by respondents.

**Figure:**
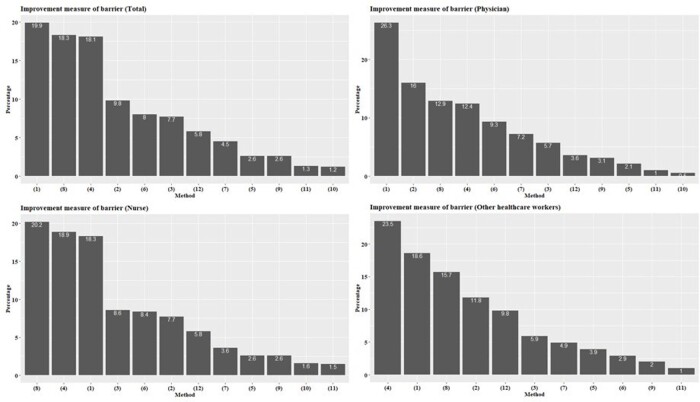

The graph shows the percentage of respondents who selected each improvement measure as their first choice. The measures include offering different types of hand sanitizers (1), sending reminders about hand hygiene timing (2), educating patients and caregivers to promote a culture of hand hygiene among staff (3), using hand hygiene campaigns to change perceptions (4), including hand hygiene results in staff performance reviews (5), providing immediate feedback on hand hygiene observations (6), conducting regular monitoring of hand hygiene practices (7), ensuring soap and paper towels are available in all hospital rooms (8), implementing a real-name system to track hand hygiene performance (9), conducting peer-to-peer assessments of hand hygiene performance (10), strengthening hand hygiene theory education (11), and providing training for different hand hygiene situations (12).

**Conclusion:**

Differences in barriers hindering HH compliance and improvement plans were identified for each group. The findings suggest that targeted interventions tailored to the specific needs of different occupational groups may be effective in improving HH compliance in healthcare settings.

**Disclosures:**

**All Authors**: No reported disclosures

